# Treatment of Pertussis

**Published:** 1892-02

**Authors:** 


### TREATMENT OF PERTUSSIS.

Dr. Hellet says that under the influence of ozone inhalations, lasting a quarter of an hour each day, the number of attacks diminishes, the general condition improves, and the appetite returns in a few days. Dr. Chibret claims that by sprinkling powdered iodoform over the pillow on which the child sleeps, the attacks of coughing are quickly arrested. Dr. Galvagno employs the following formula :

℞ Resorcin,
Antipyrin, aa gr. xv.
Acid hydrochloric, gtt. x.
Syr. simp., oz. j.
Aqua dest., oz. iij.

Of which from three to five teaspoonfuls are given daily. The average duration of cases thus treated does not exceed fifteen days. Dr. Noevius advises an infusion of thyme (100 parts to 700 parts water), to which are added 50 parts of syrup of marshmallow, and of this from a teaspoonful to a dessertspoonful is given eight or ten times a day, according to the age of the child.—*Bul. Gen. de Therapeutique*, November, 1891.

